# Physicochemical-guided design of cathelicidin-derived peptides generates membrane active variants with therapeutic potential

**DOI:** 10.1038/s41598-020-66164-w

**Published:** 2020-06-04

**Authors:** Nelson G. J. Oliveira, Marlon H. Cardoso, Nadya Velikova, Marcel Giesbers, Jerry M. Wells, Taia M. B. Rezende, Renko de Vries, Octávio L. Franco

**Affiliations:** 1 Centro de Análises Proteômicas e Bioquímicas, Programa de Pós-Graduação em Ciências Genômicas e Biotecnologia, Universidade Católica de Brasília, Brasília-DF, Brazil; 2 Physical Chemistry and Soft Matter, Wageningen University and Research, Stippeneng 4, 6708 WE Wageningen, The Netherlands; 3 Programa de Pós-Graduação em Patologia Molecular, Faculdade de Medicina, Universidade de Brasília, Brasília-DF, Brazil; 4 Host-Microbe Interactomics, Animal Science Department, Wageningen University and Research, Wageningen, The Netherlands; 5 Wageningen Electron Microscopy Centre, Wageningen University and Research, Droevendaalsesteeg 1, 6708 PB Wageningen, The Netherlands; 6 Curso de Odontologia, Universidade Católica de Brasília, Campus I, Águas Claras, Brasília, Distrito Federal Brazil; 7 S-inova Biotech, Programa de Pós-Graduação em Biotecnologia, Universidade Católica Dom Bosco, Campo Grande-MS, Brazil; 8 Programa de Pós-Graduação em Ciências da Saúde, Faculdade de Ciências da Saúde, Universidade de Brasília, Brasília-DF, Brazil

**Keywords:** Peptides, Peptides, Bacterial infection, Bacterial infection

## Abstract

The spread of multi-drug resistance and the slow pace at which antibiotics come onto the market are undermining our ability to treat human infections, leading to high mortality rates. Aiming to overcome this global crisis, antimicrobial peptides are considered promising alternatives to counter bacterial infections with multi-drug resistant bacteria. The cathelicidins comprise a well-studied class of AMPs whose members have been used as model molecules for sequence modifications, aiming at enhanced biological activities and stability, along with reduced toxic effects on mammalian cells. Here, we describe the antimicrobial activities, modes of action and structural characterization of two novel cathelicidin-like peptides, named BotrAMP14 and CrotAMP14, which were re-designed from snake batroxicidin and crotalicidin, respectively. BotrAMP14 and CrotAMP14 showed broad-spectrum antibacterial activity against susceptible microorganisms and clinical isolates with minimal inhibitory concentrations ranging from 2–35.1 μM. Moreover, both peptides had low cytotoxicity against Caco-2 cells *in vitro*. In addition, *in vivo* toxicity against *Galleria mellonella* moth larvae revealed that both peptides led to>76% larval survival after 144 h. Microscopy studies suggest that BotrAMP14 and CrotAMP14 destabilize *E. coli* membranes. Furthermore, circular dichroism and molecular dynamics simulations indicate that, in a membrane-like environment, both peptides adopt α-helical structures that interact with bilayer phospholipids through hydrogen bonds and electrostatic interaction. Thus, we concluded that BotrAMP14 and CrotAMP14 are helical membrane active peptides, with similar antibacterial properties but lower cytotoxicity than the larger parent peptides batroxicidin and crotalicidin, having advantages for drug development strategies.

## Introduction

The discovery of penicillin and streptomycin during the golden age of antibiotics heralded a revolution in medical treatment of infections. Nevertheless, the spread of antibiotic resistance has made these antibiotics ineffective against many common pathogens^1^. The overuse and/or misuse of antibiotics results in microbial resistance through a variety of mechanisms^1^. Horizontal transfer of resistance determinants within and between species, and /or DNA mutations, has led to the emergence of pathogens resistant to multiple drugs^2^, reducing our capacity to treat multi-drug resistant bacterial infections^1^. Consequently, research on the development of new strategies to control antibiotic-resistance has intensified, including the usage of antimicrobial peptides (AMPs), which represent one of the oldest innate defense mechanisms in living organisms^3–5^. The composition and biological effects of AMPs found in nature are diverse. AMPs can range in length from 2–50 amino acid residues, and have a great diversity of amino acid composition, structure and size^3^. These compounds were initially studied because of their antimicrobial activity, and it was discovered that they possess a rich spectrum of other biological activities, including immunomodulatory actions, wound healing and anticancer properties^5–9^.

AMPs are being considered as therapeutic agents but have a number of limitations, including serum stability, bioavailability, cytotoxicity and size, as well as the cost of peptide synthesis^10^. The rational (re)design of AMPs provides further possibilities to enhance their activity, increase stability, as well as specificity, and reduce their size. For example, sequence modifications of natural peptides can increase peptide helicity, hydrophobic moment and net charge, while reducing hydrophobicity^11^, all factors which are reported as crucial for cationic AMPs, including cecropins, magainin, melittin and the cathelicidins^12^. Moreover, the structural arrangements adopted by AMPs can be partially related to their amphipathic nature, which makes them able to present varied structures, including α-helices, β-sheets, coils or a mixture of all, which are important for interactions with their bacterial targets^13^.

Among AMP classes, cathelicidin related antimicrobial peptides (CRAMPs) are produced by diverse vertebrates, including mammals and snakes, and have been widely studied^14^. Cathelicidins are often expressed and secreted by epithelial cells lining animal glands or mucosa. The N-terminal domain is a highly conserved region composed of its signal peptide and the cathelin domain. In contrast, the CRAMP C-terminal domain, containing the mature peptide sequence, is highly variable in length and sequence, both interspecies and intraspecies^15^. Reptilian CRAMPs, particularly those from lizards and snakes, show broad-spectrum antimicrobial activity against a large number of bacteria, fungi and viruses^16^. In addition to their antimicrobial effect, cathelicidins have been reported to have multiple activities, including the activation of immune cells, promotion of cell proliferation, cell migration, cell survival, cytokine release, angiogenesis, chemotaxis, and wound healing, thus clearly representing a promising class of therapeutic agents^16^.

The cathelicidin AMPs originate from several sources, including snake venom^14^, which has been widely studied for its antimicrobial properties^13,17^. In the present study, two cathelicidin peptides (batroxicidin and crotalicidin) have been chosen for the physicochemical-guided design of improved variants. Batroxicidin and crotalicidin^18^ were identified in the South American pit vipers *Bothrops atrox* and *Crotalus durissus terrificus*. These peptides contain 34 amino acid residues and have exerted pronounced antimicrobial activity against diverse bacteria, with few haemolytic effects or proinflammatory effects on RAW 264.7 cells^19^. These properties suggest that batroxicidin and crotalicidin are promising candidates for antibacterial therapy. Snake AMPs, such as the snake cathelicidin NA-CATH and its smaller derivative (ATRA1), have achieved similar therapeutic potential^20,21^, suggesting it would be worthwhile to characterize these smaller batroxicidin and crotalicidin variants.

This paper describes two new synthetic peptides (BotrAMP14 and CrotAMP14) derived from the vipericidins, batroxicidin and crotalicidin^18^, which were generated by a physicochemical-guided design strategy aiming to reduce their size, as well as preserve or improve the antibacterial properties and low toxicity of their parent peptides. In addition, the modes of action of both variants on *E. coli* cells were also evaluated, and computational and experimental structural studies were performed in different biomimetic conditions to investigate structure-function relationships in these two peptides.

## Material and Methods

### Peptide re-design

Initially, the cathelicidin template sequences (batroxicidin and crotalicidin) were collected from the Uniprot knowledgebase (https://www.uniprot.org). The physicochemical characteristics for the templates and designed peptides, including pI, hydrophobicity, hydrophobic moment and net-charge, were obtained by submitting the sequences to the compute_pi algorithm^22^ (http://web.expasy.org/compute_pi/) and the Heliquest server (http://heliquest.ipmc.cnrs.fr/) helical-wheel projections^23^.

### Peptide synthesis and preparation

The peptides were purchased from Peptides 2.0 (USA) at a purity of >95%. The molecular mass for the peptides was confirmed using *Matrix Assisted Laser Desorption Ionization – Time of Flight* (*MALDI-ToF*) on a mass spectrometer Ultraflex MALDI-TOF III (Bruker Daltonics)^24^. The concentration of the designed peptides was determined using measurements of absorbance at 205, 215 and 225 nm as described by Niebergall and co-workers^25^.

### *In vitro* antimicrobial assays

Minimal inhibitory concentration (MIC) and minimal bactericidal concentration (MBC) experiments were performed for the ATCC and Clinical Isolated strains of *E. coli* 25922* K. pneumoniae 13822*, and *S. aureus* 25923. The antimicrobial activities of all the peptides were tested as previously described with minor modifications^26^. Briefly, Mueller-Hinton (Himedia) broth was used to grow the strains overnight at 37 °C. MIC measurements were performed using 1 × 10^5^ CFU.mL^−1^ and serial dilution of the peptides BotrAMP14 and CrotAMP14 starting at 50 µM. The MIC (100% inhibitory concentration) was determined after 24 h of incubation at 37 °C. The absorbance was measured in a 96-well microplate at 600 nm. Bacteria cultured only in MHB and containing the antibiotics (chloramphenicol, gentamicin and imipenem) were used as negative and positive control, respectively. MBCs were determined by plating out 10 µL of the contents of the wells where no bacterial growth was observed on MH agar plates. MBC was recorded as the lowest concentration at which no colonies were observed after 24 h incubation at 37 °C. All the measurements were performed in triplicate.

### Neutral-red (NR) *in vitro* toxicity assay

Toxicity of the peptides on Caco-2 cells, at increasing peptide concentrations, was determined using a neutral-red (NR) assay as described previously^27^. After overnight incubation of Caco-2 cells with the peptides BotrAMP14 and CrotAMP14 at concentrations from 2–35 µM, 10 μL of a 33 µg.mL^−1^ NR solution was added to the wells containing the peptide-incubated cells. After 3 h of incubation, the supernatant was removed, and the cells were washed with phosphate buffer saline (PBS). Next, 150 μL of 1% acetic acid-50% ethanol was added and shaken for 10 min at room temperature. Finally, the absorbance was measured at 540 nm and 690 nm (background absorbance) using a SpectraMax M5 microplate reader (Molecular Devices). Readings were expressed as NR uptake relative to the uptake of the cells exposed to the negative control (medium or DMSO). All the measurements were performed in triplicate.

### Galleria mellonella *in vivo* toxicity assay

*In vivo* toxicity was assayed using *Galleria mellonella* larvae^28^ in their final instar stage. The larvae were purchased (UK Waxworms Ltd, Sheffield, UK), and acclimatized in the dark at 15 °C, and used within 14 days. Only larvae weighing between 0.2 and 0.3 g were used for experiments. Larvae were injected with 20 μL of BotrAMP14 and CrotAMP14 peptide solutions, or controls in the left posterior proleg using Terumo Myjector 29 G insulin syringes (VWR International). Two negative control groups were included in every experiment; one group was not injected to control for background larval mortality (no manipulation control) and the other group (uninfected control) was injected with PBS to control for the possible effect of physical trauma on mortality. After injection, larvae were acclimatized in Petri dishes in the dark at 37 °C with 5% CO_2_ for up to 144 h post-inoculation and inspected every 24 h for survival. For each sample (non-manipulated control, water control, peptides) fifteen randomly chosen larvae were used. The peptide concentration was 10 mg.kg^−1^ of body weight. The data were expressed as % larval survival of the survival of the uninfected control. All the measurements were performed in triplicate.

### Circular Dichroism

Circular dichroism (CD) spectra were obtained using a Jasco-715 spectro-polarimeter equipped with a Peltier element for temperature control. CD spectra were obtained in the far-UV range, from 200 to 260 nm using a quartz cuvette with 0.1 cm optical path. An averaging of 20 single scans was obtained for the peptide concentration of 110 μM, for demi water, 10 mM potassium phosphate buffered saline at pH 7.4, and 30 mM of sodium dodecyl sulphate (SDS). All the spectra were fitted using the CONTIN algorithm as implemented in the DICHROWEB^29–32^ webserver, using the data basis set #7 for a quantitative interpretation of the spectra in terms of a percentage of α-helical structure.

### Molecular modeling

Initially, BLASTp^33^ was performed in order to find the best template structures for the molecular modeling of BrotAMP and CrotAMP. Further, 100 theoretical three-dimensional models were constructed using Modeller v. 9.12^33^, based on the nuclear magnetic resonance (NMR) structure of a crotalicidin isolated from venom of the rattlesnake (*Crotallus durissus*) (PDB code: 2MWT)^18^. The lowest free-energy theoretical models (DOPE score) for both peptides were then selected and used for validation procedures according to their fold (ProSA-web)^34^ and stereochemistry (PROCHECK)^35^. Structure visualization was performed in PyMOL (http://www.pymol.org).

### Molecular dynamics in water and SDS micelles

Molecular dynamics (MD) simulations for BotrAMP14 and CrotAMP14 were initially carried out in contact with an SDS micelle, according to Cardoso *et al*.^36^. The simulations were performed using the CHARMM36 force field from the computational package GROMACS v.5.0.4^37^. MD simulations in SDS were carried out in dodecahedron boxes, where both peptides were put in contact with SDS micelles constituted of 100 detergents. SDS micelles were built, and their topologies generated using the CHARM-GUI server^24^. Chloride ions were added to neutralize the systems’ charge in both simulations. The simulations were performed under 0.15 M NaCl ionic strength. Geometry of water molecules was constrained using the SETTLE algorithm^38^. Moreover, the LINCS algorithm^39^ was used to link all the atom bond lengths. Particle Mesh Ewald (PME)^40^ was used for electrostatic corrections with a radius cut-off of 1.4 nm to minimize the computational simulation time. The same radius cut-off was used for van der Waals interactions. The list of neighbors of each atom was updated every 10 simulation steps of 2 fs each. The steepest descent algorithm (50,000 steps) was applied for energy minimization. The systems underwent a normalization of temperature and pressure to 310 K and 1 bar using the velocity rescaling thermostat (NVT)^41^ and the Parrinello-Rahman barostat (NPT)^42^, respectively, for 100 ps. The systems with minimized energy and balanced temperature and pressure were submitted to MD simulations for 800 ns. MD simulations were analyzed by means of root mean square deviation (RMSD). Moreover, peptide-SDS atomic interactions were measured on PyMOL v. 1.8 (The PyMOL Molecular Graphics System, Version 1.8 Schrödinger, LLC).

### Scanning electronic microscope

Scanning electronic microscopy (SEM) was performed by the adherence of bacteria to Poly-L-Lysine-coated slides. Prior to SEM analysis, *E. coli* ATCC 25922 was grown in MHB overnight at 37 °C, and the concentration of 1 × 10^5^ CFU.mL^−1^. The bacterial cultures were centrifuged, and phosphate buffer was used to replace the MHB. The bacterial culture was increased by adding 8 µM and 2 µM for BotrAMP14 and CrotAMP14, respectively, for 5, 30 and 60 min of incubation. For control group, *E.coli* ATCC 25922 was used without peptides (time 0). Samples for SEM were prepared by leaving microscope slides coated with poly-L-lysine in 10 mL suspension of bacteria incubated with the peptides and allowing them to settle and adhere to the slides. After 60 min incubation at RT, the bacteria were fixed using 2.5% glutaraldehyde in phosphate buffer. Finally, water was removed using critical point drying: first, the samples were immersed in a graded series of increasing ethanol: 25%, 50%, 75%, and two times at 100% each for 10 min. This was followed by transferring the samples to absolute ethanol, and by critical point drying.

## Results and Discussion

### Rational design of batroxicidin and crotalicidin variants

The rational (re)design of AMPs to tune amphipathicity, degree of helicity, charge, etc.^43^, can be used to further improve their usefulness as future antimicrobial medicines. We therefore present two novel AMPs derived from snake cathelicidins. Previously, Falcao and colleagues^18^ reported the discovery of two vipericidins, named crotalicidin and batroxicidin, with very similar sequences and high antimicrobial capacity. Subsequently, Falcao and coworkers^44^ showed that just a small fragment of the C-terminal region of crotalicidin (fragment 15–34), named Ctn [15–34], was highly active against diverse microorganisms; whereas the N-terminal sequence showed low antimicrobial activity. We therefore used the peptide Ctn [15–34] as template sequence for the generation of CrotAMP14. Following the same rationale used for the peptide crotalicidin, where only its C-terminal region has been described as active against microorganisms, the C-terminal region of the peptide batroxicidin (fragment 15-34), named here as Btn [15-34], was also used as a template for the generation of another peptide called BotrAMP14.

**Figure 1:**
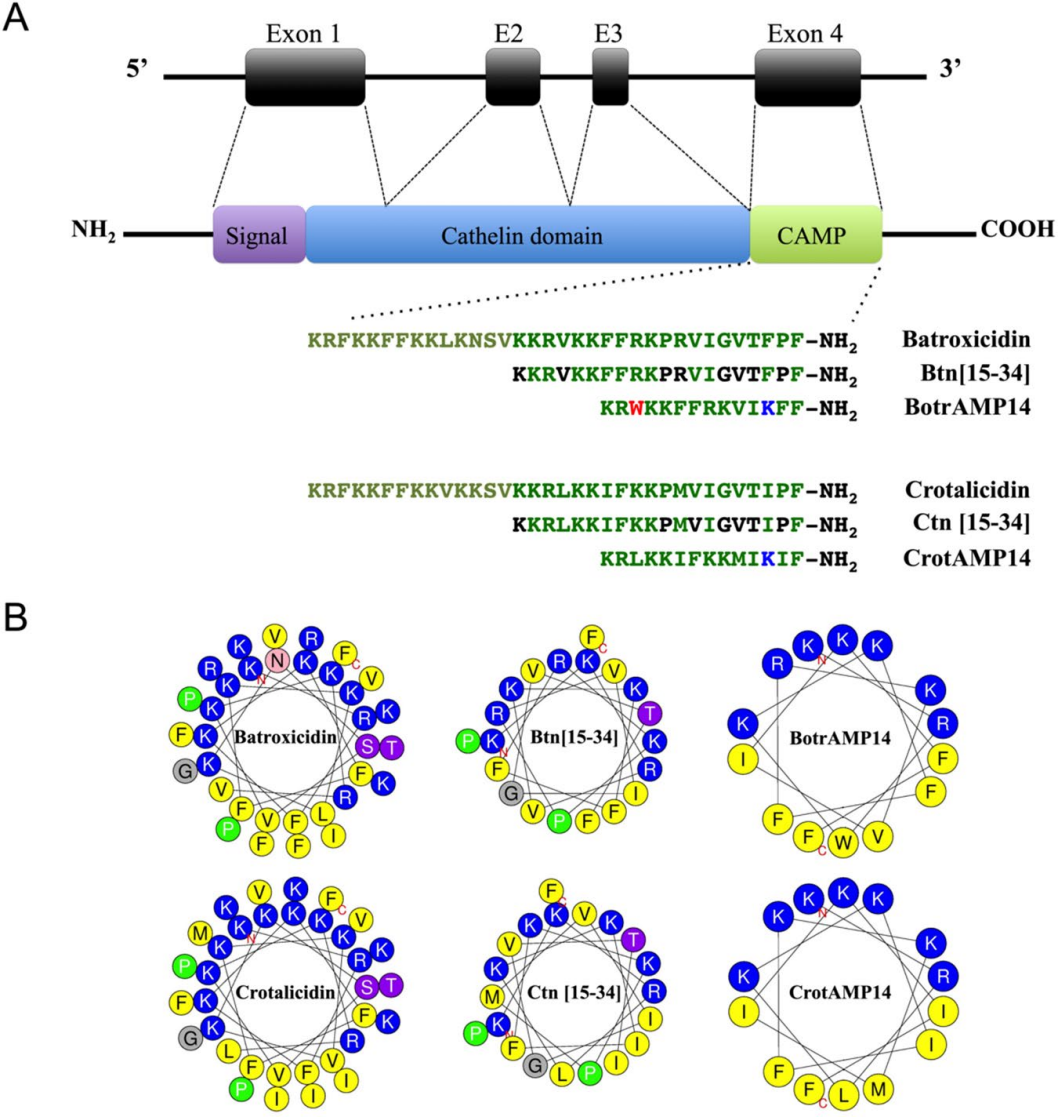
(**A**) - Domain structure of crotalicidins in general followed by the sequence of the natural occurring vipericidins (batroxicidin and crotalicidin), the sequence of the intermediate peptides (Btn[15-34] and Ctn[15-34]) and the rationally designed analogues BotrAMP14 and CrotAMP14. The green letters represent the original amino acid sequence. The black letters represent the discarded amino acid. The red letters represent hydrophobic amino acids included, and the blue letters represent the cationic amino acids included. B - Helical-wheel projections for the peptide precursors batroxicidin, crotalicidin, Btn[15-34] and Ctn[15-34], and the rational designed BotrAMP14 and CrotAMP14 (**B**).

BotrAMP14 and CrotAMP14 are 14-amino acid peptides derived from the cathelicidin subsequences: Btn[15-34] (^15^KKRVKKFFRKPRVIGVTFPF^34^) from batroxicidin; and Ctn[15-34] (^15^KKRLKKIFKKPMVIGVTIPF^34^) from crotalicidin, respectively (Fig. 1A). Initially, the parent sequences were mapped into a helical wheel diagram^23^ (Fig. 1B) to identify the cationic and hydrophobic faces of Btn[15-34] and Ctn[15-34] when folded into an α-helix. Based on these diagrams, a series of modifications in Btn[15-34] and Ctn[15-34] were carried out to reduce their size and enhanced helical amphipathicity, thus generating BotrAMP14 (KRWKKFFRKVIKFF-NH_2_) and CrotAMP14 (KRLKKIFKKMIKIF-NH_2_). The aim was to exclude amino acid residues not favorable for the electrostatic surface, while maintaining an alternation between positively charged and hydrophobic residues. The amino acid residues K^1^, V^4^, P^11^, R^12^, G^15^, V^16^, T^17^ and P^19^ in Btn[15-34] were removed from BotrAMP14, followed by the addition of tryptophan and lysine residues at position 3 and 12, respectively. Similarly, the residues K^1^, P^11^, V^13^, G^15^, V^16^, T^17^ and P^19^ in Ctn[15-34] were removed from CrotAMP14 and a lysine residue was added at position 12.

The resulting physicochemical changes in BotrAMP14 and CrotAMP14 in comparison to their parent sequences are summarized in Table 1. Both peptides were designed focusing on maintaining the high net charge of the cathelicidins’ parental sequences, alongside an increased hydrophobic moment and, mainly, the reduction of the parental sequence lengths. In a previous study developed by our group, we demonstrated that the cationic subsequence Ctn[15-34] can be inactivated through the insertion of a negatively charged propeptide sequence, as a potential prodrug strategy^45^. These peptide inactivation strategies may also be used with BotrAMP14 and CrotAMP14 peptides, as their small size and cationic properties were preserved.

**Table 1:** Physicochemical properties of BotrAMP14, CrotAMP14 and their parent peptides.

Peptides	Residues	Net charge^a^ (pH7)	pI^b^	Hydrophobicity^c^<H>	Hydrophobic moment^c^<μH>	Molecular mass
Batroxicidin	34	+16	12.50	0.207	0.473	4258.29
Crotalicidin	34	+16	12.09	0.263	0.440	4151.33
Btn[15-34]	20	+9	12.32	0.317	0.306	2478.08
Ctn[15-34]	20	+8	11.43	0.455	0.311	2371.07
BotrAMP14	14	+8	12.04	0.390	0.888	1957.46
CrotAMP14	14	+8	11.43	0.354	0.849	1820.43

^a^Determined for C-terminal amidated (NH_2_) peptides; ^b^Determined by the compute_pi algorithm (http://web.expasy.org/compute_pi/); ^c^Determined by the Heliquest server (http://heliquest.ipmc.cnrs.fr/).

Cationic AMPs from several classes (cathelicidins, defensins and magainins), are commonly modified using rational design strategies, which allow a shorter motif to be obtained, decreasing the cost of producing synthetic peptides for therapeutic applications^43^. In addition, the removal of specific residues with undesired properties may also produce peptide analogues with lower toxicity and immunostimulatory activities^43^. In the present work, the amino acid Lys (K^12^) was inserted in both redesigned variants (BotrAMP14 and CrotAMP14) with the aim of boosting electrostatic interactions with bacterial membranes and promoting antibacterial activity. Furthermore, studies have shown that lysine-rich peptides have reduced toxicity against eukaryotic cells^46^. The Trp (W^3^) was inserted in BotrAMP14 because of its ability to arrange itself more deeply into the bacterial membranes, presenting a distinct preference for the interfacial region of lipid bilayers^47^. Therefore we expected BotrAMP14 to disrupt the bacterial cell membranes more efficiently^47^. Overall, these amino acids modifications interfered at the helical wheel diagram for BotrAMP14 and CrotAMP14, resulting in clear cationic and hydrophobic residues distribution along the peptide sequence, thus favoring the amphipathicity (Table 1 and Fig. 1B).

### Antibacterial properties of BotrAMP14 and CrotAMP14

The antibacterial activity of the BotrAMP14 and CrotAMP14 peptides, as well as that of the parental template peptides Btn [15-34] and Ctn [15-34], was evaluated against several bacteria, including Gram-negative, Gram-positive susceptible strains, and resistant strains. As shown in Table 2, BotrAMP14 and CrotAMP14 exhibited a pronounced activity against both susceptible bacteria and clinical isolates. BotrAMP14 presented a stronger activity as compared to the parental Btn [15-34] for all strains tested (Table 2). For CrotAMP14 we found the same activity as for the parental peptide Ctn [15-34] for Gram-negative strains (except for *K. pneumoniae* CI 1445333), and improved activity against the Gram-positive strains.

**Table 2:** Minimal inhibitory concentration (MIC) and minimal bactericidal concentration (MBC) of the redesigned peptides, and the parental peptides against susceptible bacteria and clinical isolate strains.

Microorganisms	Gram	Btn [15-34]	Ctn [15-34]	BotrAMP14	CrotAMP14	Gentamicin	Imipenem	Chloramphenicol	Polimyxin B
		Micromole (μM)							
		MIC (MBC)							
*E. coli* ATCC 25922	—	6.2 (>50)	3.1 (25)	3.1 (50)	3.1 (25)	0,03 (1,5)	**	6.2 (6.2)	0,03 (1.5)
*E.coli* KpC+ 1812446	—	3.1 (12.5)	1.5 (6.2)	1.5 (3.1)	1.5 (3.1)	**	12.5 (>50)	**	**
*K. pneumoniae* ATCC 13822	—	12.5 (50)	6.2 (12.5)	6.2 (6.2)	6.2 (6.2)	0.03 (1.5)	**	**	**
*K. pneumoniae* CI 1445333	—	>50 (>50)	6.2 (25)	6.2 (>50)	12.5 (>50)	6.2 (12.5)	**	**	**
*S. aureus* ATCC 25923	+	>50 (>50)	>50 (>50)	12.5 (50)	12.5 (50)	0.03 (1.5)	**	**	**
*S. aureus* CI	+	25 (>50)	25 (>50)	3.1 (>50)	3.1 (>50)	0.03 (1.5)	**	**	**

The efficient antibacterial activity described here for the designed peptides BotrAMP14 and CrotAMP14 is consistent with previous works regarding the precursor CRAMPs^18,44,45^. Regarding antimicrobial activity, the higher MICs observed for the Gram-positive bacteria than for the Gram-negative bacteria tested indicate that both redesigned peptides possess a differential affinity for these types of bacterial membranes. By reducing the number of amino acid residues and increasing hydrophobic moment, the redesigned peptides had a higher MIC for the Gram-positive bacterium *S. aureus* than Gram-negative bacteria. This feature may be related to the increased helicity of the redesigned peptides BotrAMP14 and CrotAMP14 compared to the parental peptides. The propensity to form structures in amphipathic α-helices in membrane-mimicking membrane environments has been demonstrated in several studies as an essential factor for the disruptive activity of AMPs^48^. More recently, a smaller derivative of cathelicidin KP36, designated AMP (RN15), also demonstrated promising activity, mostly against Bacillus bacteria, and low hemolytic and cytotoxicity against dermal human dermal fibroblasts^49^. However, for Gram-negative strains, a slight increase of BotrAMP14 over the parental Btn [16-34] was observed. This increase may be related to the tryptophan residue presence, which could lead to an increase in membrane/peptide interaction due to tryptophan insertion into the interfacial region of the phospholipid bilayer^47^. This increase was not observed for CrotAMP14 peptide, which maintained the overall parental activity. Ultimately, the bacterial cytoplasmic membrane lipid composition seems essential for AMPs’ mechanism of action. Generally, it is constituted by zwitterionic lipid phosphatidylethanolamine (PE), and anionic lipids phosphatidylglycerol (PG) and cardiolipin (CL), which are essential for membrane organization. The lipid composition differs from one species to another. Usually Gram-positive bacteria present a high anionic lipid (PG and CL) amount, which may favor electrostatic interactions. Gram-negative bacteria show higher PE content in comparison to Gram-positive strains^50^. Such features could explain the higher increased activity against Gram-positive bacteria for the redesigned peptides over parental.

### Structural analyses

**Figure 2:**
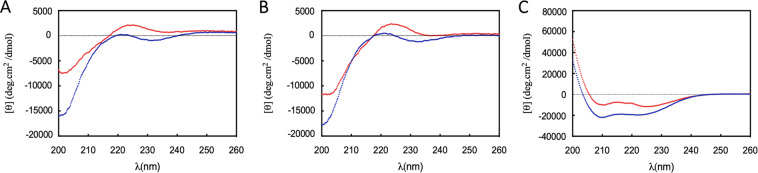
CD spectra of the peptides BotrAMP14 (red) and CrotAMP14 (Blue). Residue molar ellipticity [q] in deg.cm^2^ /dmol^−1^ is plotted versus the wavelength l in nm. Measurements were done at a peptide concentration of 0.2 mg.mL^−1^ in demi water (**A**) 10 mM K_2_HPO_4_ 50 mM Na_2_SO_4_ buffer, pH 7.4 (**B**) and 30 mM SDS (**C**).

To elucidate the secondary structure of BotrAMP14 and CrotAMP14, CD spectroscopy, molecular modeling and dynamics simulations were carried out under different experimental conditions. CD spectra were acquired in demi-water, 10 mM potassium phosphate buffer at pH 7.4 and in 30 mM SDS, and they are shown in Fig. 2. A qualitative comparison with typical CD spectra illustrates that BotrAMP14 and CrotAMP14 spectra are characteristic of predominantly random coil configurations in demi-water and in 10 mM potassium phosphate buffer (Fig. 2A,B). In contrast, CD spectra of BotrAMP14 and CrotAMP14 indicated substantial α-helical configuration in a membrane-like environment (SDS) (Fig. 2C). For a more quantitative assessment, we used the CONTIN algorithm to fit the CD spectrum and extract percentages of the α-helical secondary structure of the peptides (2A-B). The results show BotrAMP14 has an estimated percentages of random coil structure in demi water and 10 mM potassium phosphate buffer of ~38% and ~61%, respectively.

For CrotAMP14, the estimated percentages of random coil were very similar in demi-water and buffer (i.e. 48% and ~66% respectively). However, the dominant α-helical content of secondary structure in 30 mM SDS was ~65% and ~69%, respectively, for BotrAMP14 and CrotAMP14. Similarly, Chen and coworkers^51^ also demonstrated that the smaller oligopeptide of 15 amino acid residues (^1^VKRFKKFFRKLKKSV^15^) derived from the cathelicidin BF-30 (Bf-CRAMP), revealing that an oligopeptide of 15 amino acid residues (^1^VKRFKKFFRKLKKSV^15^) maintained its minimal α-helical structure required for an antimicrobial activity. Another study on cathelicidins focused on the use of short synthetic peptides of sequences incorporated into *Ophiophagus hannah* peptides (Oh-CRAMP and Oh-CATH) and distinct antimicrobial activity and hemolysis were demonstrated relative to human erythrocytes in comparison with their original parent sequences^52^.

**Figure 3:**
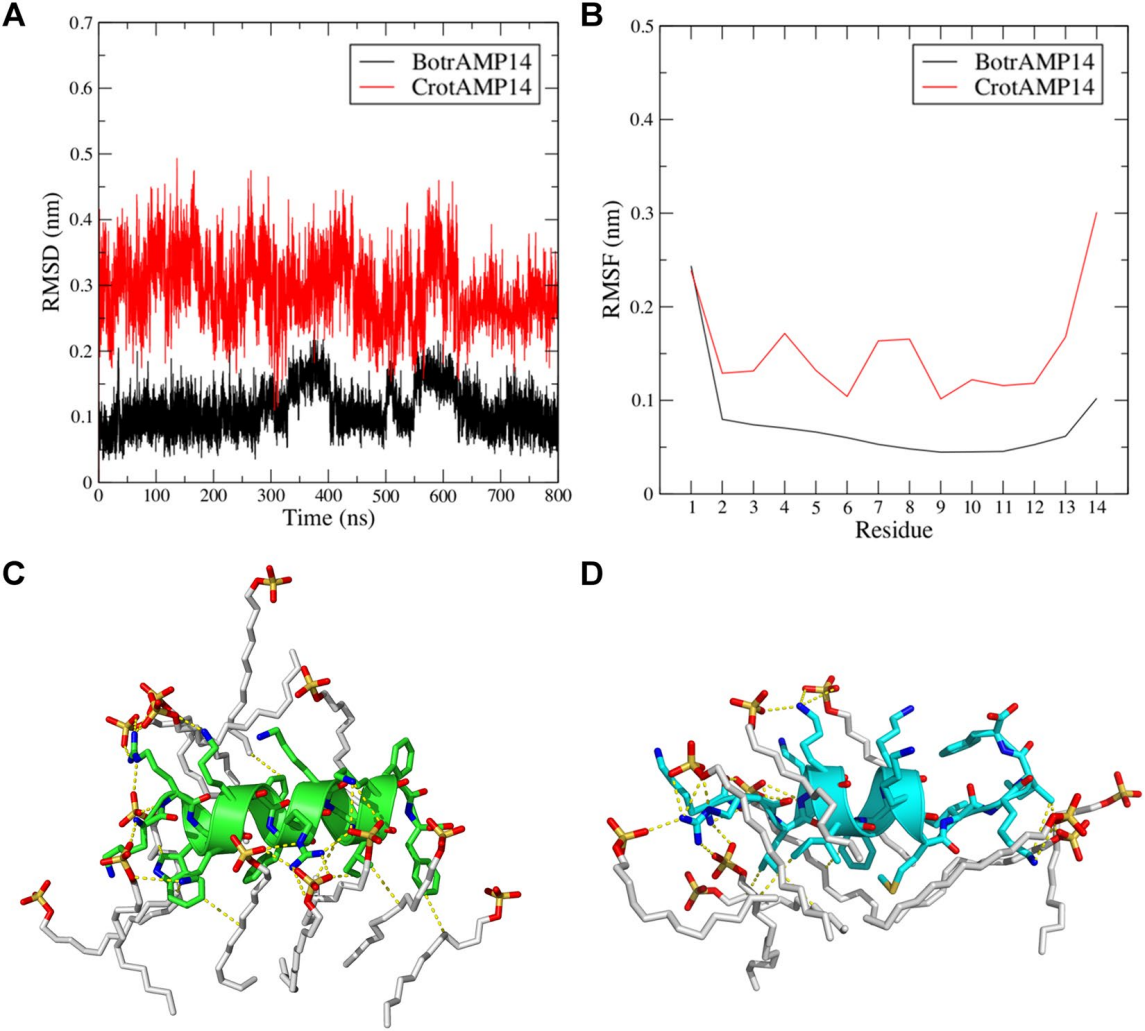
MD simulation for BrotAMP14 and CrotAMP14 peptides in the presence of SDS micelles. MD simulations were analyzed by determining root mean square deviations (RMSD) (**A**) and root mean square fluctuation (RMSF) (**B**). The three-dimensional structures of BrotAMP14 (**C**) and CrotAMP14 (**D**) were visualized after 800 ns of MD simulations, indicating the presence of a higher α-helix content for BrotAMP14 as compared with CrotAMP14. White sticks represent selected SDS molecules that are involved in stabilizing the peptide secondary structure.

MD simulations were performed for similar conditions as those used in the CD experiments. MD simulations in the presence of SDS micelles showed that the SDS clearly promoted α-helical conformations for both BotrAM14 and CrotAMP14 (Fig. 3C,D). Also, supporting the results of the CD experiments, the two peptides differ in overall helical chemical contents (Fig. 3A,B). According to the RMSD analysis CrotAMP14 showed ~3-fold higher deviations (~0.45 nm) in its trajectory when compared to BotrAMP14 (~0.15 nm) (Fig. 3A). This was also reflected in the fluctuation per residue observed for these peptides, where higher RMSF values were observed for CrotAMP14 (Fig. 3B).

Our SEM results suggest that both BotrAMP14 and CrotAMP14 directly attach to bacterial surfaces and trigger membrane perturbation events in Gram-negative bacteria. Interestingly, as for our simulations in the presence of SDS micelles, CrotAMP14 deviated and fluctuated more during the entire simulation (Fig. 3A). A total of 16 atomic interactions were predicted for the molecular complex BotrAMP14/SDS. These interactions involved the residues K^1^, R^2^, W^3^, K^4^, K^5^, F^6^, R^8^, V^10^, I^11^, K^12^ and F^14^ in BotrAMP14 (Supplementary Table 1). Furthermore, 13 interactions were predicted for the complex CrotAMP14/SDS. These interactions involve the residues K^1^, R^2^, L^3^, K^4^, K^5^, I^6^, F^7^, K^12^ and I^13^ in CrotAMP14 (Supplementary Table 2). These data clearly indicate the relevance of the positively charged residues of both peptides for initial electrostatic and hydrogen bond interactions with the sulfate groups of the SDS molecules.

### Membrane interaction analyses

**Figure 4:**
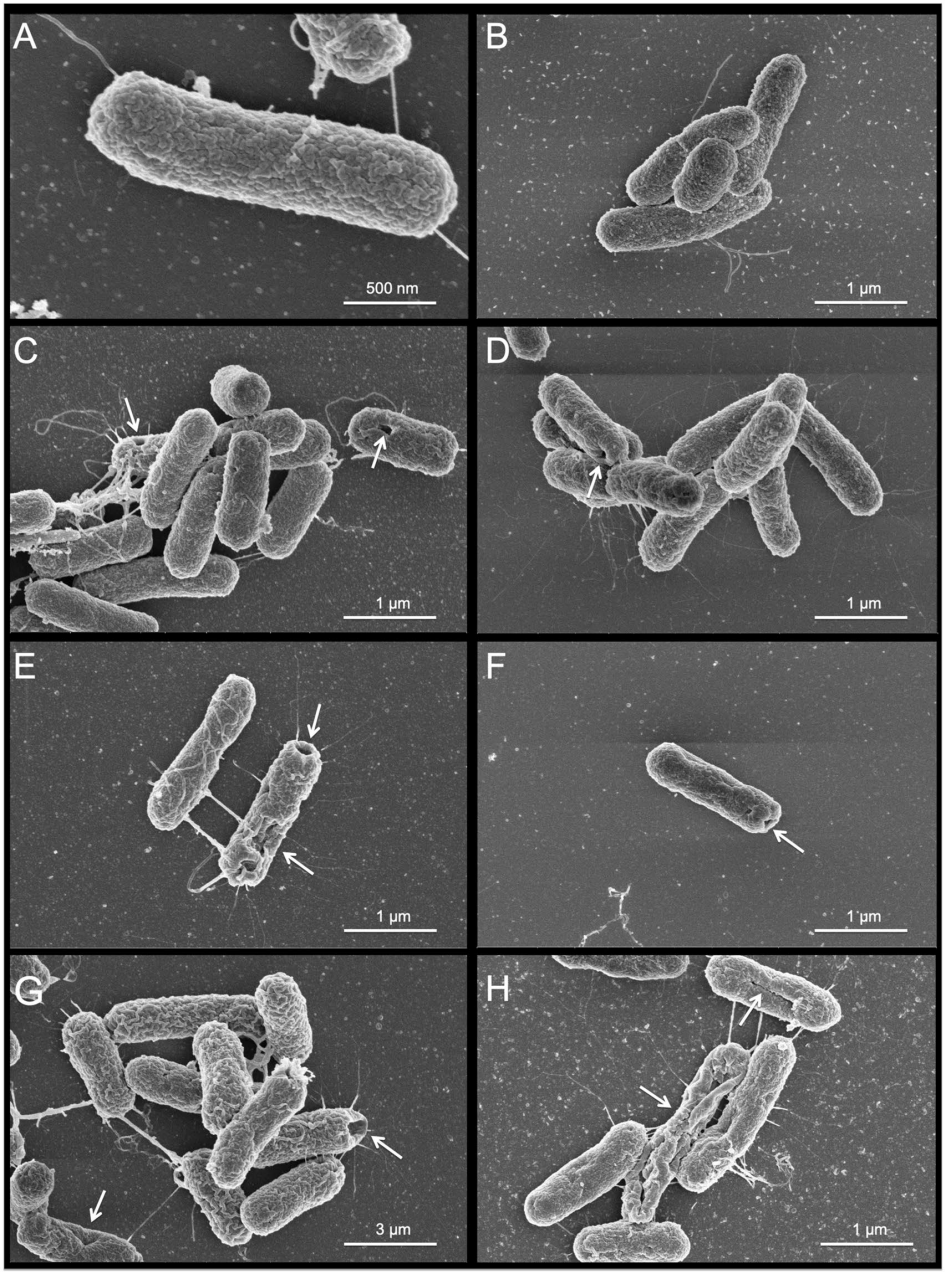
Scanning electronic microscope (SEM) high-resolution images of *E. coli* ATCC 25922 in the presence of BotrAMP14 (8.1 μM) and CrotAMP14 (2.2 μM) peptides after 0 min (**A,B**), 5 min (**C,D**), 30 min (**E,F**) and 60 min (**G,H**) of incubations. Arrows indicate cell damage. Panels A, C, E, and G represent the treatments with BotrAMP14. Panels B, D, F, and H represent the treatments with CrotAMP14 peptide.

Scanning electron microscope (SEM) analysis was performed to clarify possible morphological alterations at MIC concentration for BotrAMP14 and CrotAMP14 at different incubation times. The high-resolution images showed that both peptides induced similar damage to *E. coli* ATCC 25922 bacterial cells (Fig. 4). At time 0 h (control), no damage in the bacterial cells (Fig. 4A,B) was observed. However, after 5 min of exposure to the peptides, bacterial outer membrane damage, including holes and fissures, was clearly visible (Fig. 4C,D). The number of these “holes” increased after 30 min incubation (Fig. 4E,F). After 1 h of incubation larger-scale damage to the cells was apparent (Fig. 4G,H). The results achieved by SEM corroborate the MD simulations. The peptide/membrane interactions using SDS micelles were used to check the interaction between the peptides under membrane-mimicking conditions, indicating that peptide/membrane interactions in both cases are driven by hydrogen bond, hydrophobic interaction, and salt bridge (electrostatic interactions) between the BotrAMP14 and CrotAMP14 peptides, and the SDS micelles groups (sulfate and acyl chain). According to Pérez-Peinado and coworkers^53^ cationic and α-helical peptides in crotalicidin and its analogue Ctn [15-34] would accumulate and interact with the negative charges on the bacterial surface. The results of flow cytometry confirmed that both crotalicidin and Ctn [15-34] permeabilize the bacterial cell membrane in different ways, suggesting their precise mechanisms of action differ^53^. Based on results obtained by Perez-Peinado and coworkers^53^ we inferred that both the redesigned peptides used in our study can act in a similar way to those described for crotalicidin and Ctn [15-34]. Indeed, the peptides BotrAMP14 and CrotAMP14 maintained many of the characteristics presented by original peptides (i.e. Btn [15-34] and Ctn [15-34]), even after their reduction in size.

### Cytotoxicity of BotrAMP14 and CrotAMP14

**Figure 5:**
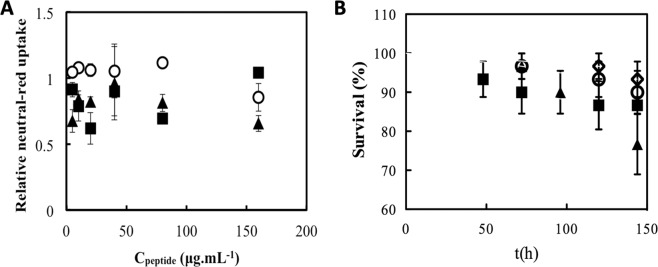
Toxicity of the peptides BotrAMP14 and CrotAMP14 on Caco-2 cells. *In vitro* neutral red uptake assay using Caco-2 cells. Viability as compared to untreated Caco-2 cells as a function of peptide concentration (**A**). Empty circle: PBS buffer control, filled square: BotrAMP14, filled triangle: CrotAMP14. **(B)** *In vivo* toxicity of BotrAMP14 and CrotAMP14 on *Galleria mellonella* larvae represented by percentage of surviving larvae as a function of time. Empty circle: H_2_O control, empty diamond: non-manipulated, filled square: BotrAMP14, filled triangle: CrotAMP14. All the experiments were performed in triplicate.

To be used as drugs, antibacterial compounds must have high selectivity to the pathogens at therapeutic concentrations in the body and must lack cytotoxicity to the host cells. Usually, the cytotoxic properties associated with both naturally occurring and rationally designed AMPs represent a bottleneck in the treatment of infections using these molecules. In this context, the toxic effects of BotrAMP14 and CrotAMP14 on mammalian cells were initially investigated *in vitro* using Caco-2 cells. Neutral red uptake (NRU) assay was used to determine the viability of mammalian Caco-2 cells as a function of peptide concentration. We observed that cells incubated with BotrAMP14 showed 100% viability at the maximum concentration tested (88 μM (Fig. 5A). At this same concentration, however, the CrotAMP14 peptide was cytotoxic, reducing cell viability to 60% (Fig. 5A). Caco-2 cells can be widely used to test the intestinal permeability and toxicity for several drugs^54^. The lack of toxicity of BotrAMP14 and CrotAMP14 against Caco-2 cells is very promising and can be used for future intestinal permeability assays, since antibiotics may lead to an imbalance of the microbiota, which is important when considering the inclusion of new drugs on the market.

### Toxicity of BotrAMP14 and CrotAMP14 to Galleria mellonela larvae

Infectivity trials and toxicity testing in rodents are important requisites for the use of drug candidates in humans. However, trials in rats and mice are expensive and there are ethical considerations. *G. mellonella* (greater wax moth) larvae are a potential alternative as they have evolutionarily conserved immunity consisting of both cellular and humoral defenses^55^. *In vivo* experiments were performed using greater wax moth *G. mellonella* larvae for a period of 144 h, at a peptide concentration of 10 mg.kg^−1^ of body weight (Fig. 5B). Survival curves indicated that both BotrAMP14 and CrotAMP14 have low toxicity for the larvae. For BotrAMP14, we observed 86.6% of larval survival after 144 h of experiment. The peptide CrotAMP14 was slightly more toxic, resulting in 76.6% larval survival after 144 h (Fig. 5B). Latour and coworkers^21^ showed the antimicrobial activity of peptides derived from *Naja atra*, composed of the ATRA motif, i.e, KR(F/A)KKFFKK(L/P)K, with a trivial toxicity against erythrocytes^21^. Contrary to what was demonstrated here, Wang and coworkers^56^ showed that derivative crotalicidin EVP50 presented the highest toxicity activity against zebrafish larvae in comparison to the original vipericidin sequences. On the other hand, other studies performed with analogues/derivatives of cathelicidins reported that these peptides had low toxicity and hemolytic activity^51^.

In conclusion, it was possible to observe that the physicochemical-guided design of BotrAMP14 and CrotAMP14 preserved the antibacterial and non-toxic potential of their parent sequences despite their smaller size, suggesting the applicability of these variants as new drug candidates. We also concluded that both peptides act on Gram-negative bacteria through a membrane destabilization mechanism, which is mainly driven by electrostatic interactions and hydrogen bonding between the N-terminus region of these peptides and anionic membranes, leading to peptide anchoring and insertion. Overall, BotrAMP14 and CrotAMP14 appear as suitable candidates for further drug development, especially for the treatment of resistant Gram-negative bacteria-associated infections.

## Supplementary information

Supplementary Information.
