# Supplementary material for: Physicochemical-guided design of cathelicidin-derived peptides generates membrane active variants with therapeutic potential
Source: Sci Rep. 2020 Jun 4;10:9127. doi: 10.1038/s41598-020-66164-w (PMC7272458; doi:10.1038/s41598-020-66164-w)
Supplement: Supplementary file 1 — Supplementary Information. [file 41598_2020_66164_MOESM1_ESM.docx]

**Physicochemical-guided design of cathelicidin-derived peptides generates membrane active variants with therapeutic potential**

Nelson G. O. Júnior^1,2,3^, Marlon H. Cardoso^1,3,7^, Nadya Velikova^4^, Marcel Giesbers^5^, Jerry M. Wells^4^, Taia M. B. Rezende^1,6,8^, Renko de Vries^2^ and Octávio L. Franco^1,3,7*^

^1^Centro de Análises Proteômicas e Bioquímicas, Programa de Pós-Graduação em Ciências Genômicas e Biotecnologia, Universidade Católica de Brasília, Brasília-DF, Brazil

^2^Physical Chemistry and Soft Matter, Wageningen University and Research, Stippeneng 4, 6708 WE Wageningen. The Netherlands

^3^Programa de Pós-Graduação em Patologia Molecular, Faculdade de Medicina, Universidade de Brasília, Brasília-DF, Brazil

^4^Host-Microbe Interactomics, Animal Science Department, Wageningen University and Research, Wageningen. The Netherlands

^5^Wageningen Electron Microscopy Centre, Wageningen University and Research, Droevendaalsesteeg 1, 6708 PB Wageningen. The Netherlands

^6^Curso de Odontologia, Universidade Católica de Brasília, Campus I, Águas Claras, Brasília, Distrito Federal, Brazil

^7^S-inova Biotech, Programa de Pós-Graduação em Biotecnologia, Universidade Católica Dom Bosco, Campo Grande-MS, Brazil

^8^Programa de Pós-Graduação em Ciências da Saúde, Faculdade de Ciências da Saúde, Universidade de Brasília, Brasília-DF, Brazil

Nelson Gomes de Oliveira Júnior: juroyal@gmail.com

Marlon Henrique Cardoso: marlonhenrique6@gmail.com

Nadya Velikova: nadya.velikova.wur@gmail.com

Marcel Giesbers: marcel.giesbers@wur.nl

Jerry Wells: jerry.wells@wur.nl

Taia Maria Berto Rezende: taiambr@gmail.com

Renko de Vries: renko.devries@wur.nl

Octávio Luiz Franco: ocfranco@gmail.com

**Supplementary Information**

| **Supplementary Table 1**. Predicted atomic interaction between BrotAMP14 after 800 ns of MD simulations in contact with an SDS micelle. | | | | | | | |
| --- | --- | --- | --- | --- | --- | --- | --- |
| **BrotAMP14** | | |  | **SDS micelle** | |  | |
| **Residue** | **Position** | **Atom** | **Distance (Å)** | **Group** | **Atom** | **Interaction** | |
| **Lys** | 1 | N | 2.7 | Sulfate | OS2 | HB | |
| **Arg** | 2 | N | 2.7 | Sulfate | OS3 | HB | |
| **Arg** | 2 | NE | 3.0 | Sulfate | OS2 | HB | |
| **Arg** | 2 | NH1 | 2.9/3.0/3.2 | Sulfate | OS1/OS3/OS4 | SB | |
| **Arg** | 2 | NH2 | 2.7 | Sulfate | OS4 | SB | |
| **Trp** | 3 | CD1/CD2/CZ3 | 3.5/3.6 | Acyl chain | C3/C6/C12 | H | |
| **Lys** | 4 | NZ | 3.6 | Sulfate | OS1 | SB | |
| **Lys** | 5 | NZ | 3.0 | Sulfate | OS1 | SB | |
| **Phe** | 6 | CD1/CE2 | 3.6 | Acyl chain | C10/C12 | H | |
| **Arg** | 8 | NE | 2.7 | Sulfate | OS2 | HB | |
| **Arg** | 8 | NH1 | 2.7 | Sulfate | OS3/OS4 | SB | |
| **Arg** | 8 | NH2 | 2.7/2.8/3.5 | Sulfate | OS1/OS2 | SB | |
| **Val** | 10 | CG1/CG2 | 3.5/3.6 | Acyl chain | C9/C12 | H | |
| **Ile** | 11 | CD/CG2 | 3.6 | Acyl chain | C6/C12 | H | |
| **Lys** | 12 | NZ | 3.6 | Sulfate | OS3/OS4 | SB | |
| **Phe** | 14 | CE1 | 3.6 | Acyl chain | C4 | H | |
| **HB: hydrogen bond; SB: saline bond (electrostatic interaction); H: hydrophobic interaction.** | | | | | | |  |

| **Supplementary Table 2**. Predicted atomic interaction between CrotAMP14 after 800 ns of MD simulations in contact with a SDS micelle. | | | | | | | |
| --- | --- | --- | --- | --- | --- | --- | --- |
| **BrotAMP14** | | |  | **SDS micelle** | |  | |
| **Residue** | **Position** | **Atom** | **Distance (Å)** | **Group** | **Atom** | **Interaction** | |
| **Lys** | 1 | N | 2.7/2,8 | Sulfate | OS2 | HB | |
| **Lys** | 1 | O | 3.6 | Sulfate | OS3 | HB | |
| **Arg** | 2 | NE | 2.9 | Sulfate | OS4 | SB | |
| **Arg** | 2 | NH1 | 2.9 | Sulfate | OS4 | SB | |
| **Arg** | 2 | NH2 | 2.7/3.1/3.3 | Sulfate | OS1/OS2/OS4 | SB | |
| **Leu** | 3 | N | 2.9 | Sulfate | OS4 | HB | |
| **Leu** | 3 | CD1/CD2 | 3.6 | Acyl chain | C2/C8 | H | |
| **Lys** | 4 | N | 3.0/3.5 | Sulfate | OS1/OS4 | HB | |
| **Lys** | 5 | NZ | 3.3/3.6 | Sulfate | OS2/OS3/OS4 | SB | |
| **Ile** | 6 | CD | 3.6 | Acyl chain | C2 | H | |
| **Phe** | 7 | CE | 3.6 | Acyl chain | C7 | H | |
| **Lys** | 12 | NZ | 3.4 | Sulfate | OS3 | SB | |
| **Ile** | 13 | CG2 | 3.6 | Acyl chain | C2/C4 | H | |
| **HB: hydrogen bond; SB: saline bond (electrostatic interaction); H: hydrophobic interaction.** | | | | | | |  |
